# HIV testing and the HIV care continuum among sub-Saharan African men who have sex with men and transgender women screened for participation in HPTN 075

**DOI:** 10.1371/journal.pone.0217501

**Published:** 2019-05-31

**Authors:** Theo G. M. Sandfort, Karen Dominguez, Noel Kayange, Arthur Ogendo, Ravindre Panchia, Ying Q. Chen, Wairimu Chege, Vanessa Cummings, Xu Guo, Erica L. Hamilton, Michael Stirratt, Susan H. Eshleman

**Affiliations:** 1 HIV Center for Clinical and Behavioral Studies, Columbia University, New York, NY, United States of America; 2 Desmond Tutu HIV Centre, UCT Medical School, Cape Town, South Africa; 3 Johns Hopkins Medical College, Malawi, Blantyre, Malawi; 4 Kenya Medical Research Institute (KEMRI) CDC, Kisumu, Kenya; 5 Perinatal HIV Research Unit, Univ. of the Witwatersrand, Soweto HPTN CRS, Soweto, South Africa; 6 Vaccine and Infectious Disease Division, Fred Hutchinson Cancer Research Center, Seattle WA, United States of America; 7 National Institute of Allergy and Infectious Disease, National Institutes of Health (NIH), Bethesda MD, United States of America; 8 Department of Pathology, Johns Hopkins University School of Medicine, Baltimore, MD, United States of America; 9 Science Facilitation Department, FHI, Durham, NC, United States of America; 10 National Institute of Mental Health, Division of AIDS Research, Bethesda MD, United States of America; University of Pennsylvania, UNITED STATES

## Abstract

Throughout the world, men who have sex with men (MSM) are at increased risk for HIV infection compared to heterosexual men. Little is known about awareness of HIV infection and other gaps in the HIV care continuum for MSM, especially in sub-Saharan Africa (SSA). This information is urgently needed to address the HIV epidemic in this population. This study assessed gaps in the HIV care continuum among persons screened for participation in a multi-country prospective study that evaluated the feasibility of recruiting and retaining MSM for HIV prevention studies in SSA (HIV Prevention Trials Network (HPTN) 075, conducted in four cities in Kenya, Malawi, and South Africa). Participants were recruited using site-specific strategies, that included outreach and informal networks. Transgender women (TW) were eligible to participate. During screening, 601 MSM and TW were tested for HIV infection and asked about prior HIV testing, HIV status, engagement in care, and HIV treatment. Viral load testing and retrospective antiretroviral (ARV) drug testing were performed for HIV-infected participants. Most participants (92.2%) had a prior HIV test; 42.1% were last tested >6 months earlier. HIV prevalence was 30.4%. HIV infection was associated with older age and identifying as female or transgender; 43.7% of the HIV-infected participants were newly diagnosed, especially younger persons and persons with a less recent HIV test. Almost a third of previously-diagnosed participants were not linked to care. Most participants (88.7%) in care were on ARV treatment (ART). Only about one-quarter of all HIV-infected participants were virally suppressed. These findings demonstrate substantial prevalence of undiagnosed HIV infection and sub-optimal HIV care engagement among MSM and TW in SSA. Increased HIV testing frequency and better linkage to care represent critical steps in preventing further HIV transmission in this population. Once in care, gaps in the HIV care continuum appear less critical.

## Introduction

As elsewhere in the world, men who have sex with men (MSM) in sub-Saharan Africa (SSA) are at increased risk for HIV infection compared to heterosexual men [1–7]. The observed HIV prevalence among MSM in SSA ranges from 7.8% in Khartoum, Sudan to 49.5% in Johannesburg, South Africa [1–3, 8–14]. Even though most countries in SSA have generalized HIV epidemics, HIV prevalence in men MSM, estimated to be 18% overall, is substantially higher (odds ratio = 3.8) than that of adult men in general [15, 16]. Furthermore, studies of the modes of HIV transmission show that while MSM constitute a relatively small proportion of the general population, their contribution to HIV incidence is substantial [17–20]. Modelling studies strongly suggest that addressing the HIV epidemic among MSM will substantially impact the general HIV epidemic in SSA [21]. Information about HIV infection in transgender women (TW) in SSA is available from studies of MSM that also assessed gender identity. Using data from eight countries in SSA, Poteat et al. [22] concluded that the adjusted odds of HIV infection for trans women were 2.2 times greater than those for cis-gender MSM. Other studies reported similar findings [23, 24].

A critical step in addressing the HIV epidemic in MSM and TW (MSM&TW) is to increase awareness of HIV status through HIV testing. Persons who know that they are HIV uninfected can benefit from risk reduction counseling and be referred for pre-exposure prophylaxis (PrEP), which is becoming more available in select settings in SSA. Persons who know that they are HIV infected can be referred for care and HIV treatment. Many countries in SSA now recommend starting antiretroviral treatment (ART) as soon as possible for all HIV-infected persons, regardless of age, CD4 cell count, clinical stage, or comorbidities. Early ART initiation has personal health benefits and limits transmission of HIV to others [25, 26].

Little is known about the awareness of HIV infection status in MSM TW in SSA. Early studies among MSM suggested that many did not know their status [12, 14]. There is also limited data available about other gaps in the HIV care continuum for this population. We evaluated these issues using data collected from persons who were screened of for participation in the HIV Prevention Trials Network (HPTN) 075 study. The overall aim of this multi-country prospective study was to evaluate the feasibility of recruiting and retaining MSM&TW for HIV prevention studies in SSA. In this report, we explored factors associated with prior HIV testing, HIV infection, awareness of HIV positive status, engagement in care, ART, and viral suppression. Findings from this study help identify critical gaps in the HIV care continuum among MSM&TW in SSA.

Legal and social factors impact the HIV/AIDS epidemic in MSM&TW in SSA [27]. Same-sex sexuality is criminalized in most SSA countries, including Kenya and Malawi, with sentences ranging up to the death penalty [28]. Compared to other parts of the world, countries in SSA are among the least accepting of same-sex sexuality [29]. Experiences with homophobia, including violence and blackmail, are well-documented among this population [12, 30]. While sexual orientation is protected by the constitution in South Africa [31, 32], same-sex sexuality remains highly stigmatized, with the social acceptance of same-sex sexuality among the lowest in the world [33]. Stigma associated with same-sex sexuality and gender diversity impacts HIV risk and limits access to HIV prevention and care. In prior studies, stigma was associated with a lack of access to condoms and lubricants, HIV testing and ART [27, 34–37]. On a larger scale, stigma has been associated with country-level investment in HIV-related services for sexual and gender minorities [35].

## Methods

### Study setting

The HPTN 075 study was conducted in four cities in three African countries (Kisumu, Kenya; Blantyre, Malawi; Cape Town and Soweto, South Africa).

### Study population

This study evaluated MSM&TW who were screened for participation in HPTN 075. Included in the current analysis were persons who were 18 to 44 years of age, were assigned male sex at birth, ever had sex with a man, and had two concordant rapid HIV test results at screening. HIV-infected and HIV-uninfected persons were eligible for participation. Participation was open to persons who were assigned male sex at birth but identified as female or transgender. The reason for including TW in this study was based on the experience that some TW in SSA identify with or see themselves as part of gay communities [38], and that HPTN 075 was designed to be inclusive and respect communities. Furthermore, inclusion of TW would allow for the collection of exploratory information about this critical high-risk population. In this report, MSM&TW refers to all persons in the study population, including MSM and persons who identified as female or transgender. More than 1 in 5 persons who screened for the study identified as female or transgender. Of the 624 persons screened for HPTN 075, 601 were included in this study.

### Study procedures

Recruitment procedures in HPTN 075 varied among study sites to allow for (a) development of strategies in consultation with the community; (b) customization to local circumstances; and (c) local adjustments of strategies if recruitment outcomes lagged. Recruitment strategies were developed in collaboration with local communities of MSM&TW, and involved peer outreach, participant referral, indirect recruitment (outreach via venues and social media), and referral through key figures in the community.

Screening study procedures included, collection of locator information, a short behavioral survey administered by trained interviewers, a blood draw, HIV counselling and testing, and risk reduction counseling (including provision of condoms and lubricant). HIV testing included two rapid tests. Those with reactive test results had an additional study visit that included repeat HIV testing. HIV-infected participants were referred for HIV care per local guidelines.

The short survey started with questions about the persons’ date of birth and current age. To screen out cis-gender women and determine current gender identification, persons were asked what sex they were assigned at birth. The question about current gender identification was introduced as follows: “The next question asks about gender. Gender is the social part of being male or female. It relates to your self-identity. When I ask about gender, I am asking about whether you regard yourself to be male, female, transgender female, or if you identify yourself in an additional category.” In line with recommended procedures for identifying persons as transgender [39–41], persons who were assigned male sex at birth who did not identify as male were classified as transgender. For persons classified as transgender, the interviewer asked the person’s gender pronoun of choice (‘he/him’ vs ‘she/her’), and documented this in the person’s chart notes, to ensure that these individuals would be appropriately addressed at subsequent visits. Immigration status was determined by asking persons for the name of the country where they were born; if this was a different country than the site where they were screened, persons were classified as immigrants.

Questions about sexual behavior were introduced with the following statement: “The next questions are about your sexual behavior. Please remember that this information is completely confidential. You may skip any question. However, we need some of this information to determine if you are eligible to participate in the study. If you choose to skip a question, it might make you ineligible to participate. Do you have any questions before we begin?” The first question that followed asked: “In your life, have you had sex with women only, with men only, or with both?” Subsequently, persons were asked: “When is the last time that you had sex with somebody?” and “In the past three months, have you had sex with men or with women?” To assess whether persons had had anal sex, the interviewer read the following instruction: “Men can have different kinds of sex with men. Men can masturbate each other. Men can have oral sex; that is when one man takes the penis of another man in his mouth. And men can have anal sex; that is when one man puts his penis in the anus of the other man.” Subsequently, persons were asked: “In the past three months, which kinds of sex have you had with men? You may indicate more than one.”

All participants were asked about prior HIV testing experiences. First, persons were asked whether they ever had been tested for HIV. Participants who reported having had a prior HIV test were subsequently asked about the number of prior HIV tests, the time since their last HIV test, and their last HIV test result. Participants who reported having had a prior positive HIV test were asked about their engagement in HIV care. Participants who reported that they were in care were asked if they had ever been prescribed ARV drugs.

The institutional review board or ethics committee at each participating site approved the protocol, and participants provided written informed consent except at site per direction of the ethics committee.

### Laboratory methods

Additional retrospective laboratory testing was performed at the HPTN Laboratory Center (Johns Hopkins Univ. School of Medicine, Baltimore, MD). This included confirmation of HIV status, ARV drug testing, and viral load testing. ARV drug testing and HIV viral load testing were only performed for participants who tested positive for HIV infection at the screening visit. ARV drug testing was performed using a qualitative assay that detects 20 ARV drugs in five drug classes [42]. Viral load testing was performed using the RealTime HIV-1 Viral Load Assay (Abbott Molecular, Abbott Park, IL).

### Statistical analyses

Participants were classified as HIV-infected or HIV-uninfected based on the results of HIV testing using samples collected at the screening visit. Data from both self-report and retrospective ARV drug testing were used to classify HIV-infected participants as: (1) having had a prior HIV test vs. not having had a prior HIV test; (2) being in care vs. not in care; and (3) being on ART vs. not on ART. Detection of ARV drugs in study samples was considered to indicate prior HIV testing, care engagement, and ART, even if these were not reported to study staff. Data from self-report alone was used to assess the frequency of prior HIV testing and the time since the last HIV test. Univariate and multivariate logistic regression analyses were used to compare (1) persons who never had an HIV test with those who had one or more HIV tests; (2) persons who tested HIV positive with those who tested HIV negative; and (3) persons who were newly diagnosed with those who were previously diagnosed. Co-variates included age, immigration status, sexual history, HIV testing practices, and current sexual activity. Multivariate models included variables that had an odds ratio with a p-value < 0.1 in the univariate analysis, as a conservative cut-off point. For one model a p-value < 0.05 was used to prevent overspecification. For some multivariate analyses, participants were excluded because 'testing history' and 'testing frequency' were not known (these data were only collected for participants who reported having had a prior HIV test).

### Ethical clearances

This study was approved by local and international institutional review boards, including Johns Hopkins University and College of Medicine Ethics Research Committee (COMREC) for the Malawi site; Kenya Medical Research Institute (KEMRI) for the Kenya site; University of the Witwatersrand (Wits) for the Soweto site; the University of Cape Town for the Cape Town site; and the New York State Psychiatric Institute.

## Results

### Demographics, testing behavior, and HIV prevalence

Between June 2015 and July 2016, 624 persons were screened for participation in HPTN 075. Twenty-three persons were excluded because they did not meet inclusion criteria for the current analysis. Exclusion was largely due to never having had sex with men (9); discordant test results (6); and being too young (4). Four persons were unwilling to undergo HIV testing throughout the study and to receive these test results; these individuals had no HIV test result at screening. Among the remaining 601 MSM&TW persons who were eligible for this study, the median age was 23 years (interquartile range [IQR]: 20–28 years). One hundred thirty-six participants who identified as female or transgender were classified as TW (22.6%; 136/601, Table 1). Over half of the participants (57.6%; 346/601) reported ever having had sex with women (Table 1).

**Table 1:** Characteristics of MSM and TW screened for participation in HPTN 075 (N = 601).

Variables	Total N (%)	HIV Prevalence (%)
**Age (years)**		
18–20	152/601 (25.3%)	35/152 (23.0%)
21–25	237/601 (39.4%)	66/237 (27.8%)
26–44	212/601 (35.3%)	82/212 (38.7%)
**Gender identity**		
Male	465/601 (77.4%)	125/465 (26.9%)
Transgender	136/601 (22.6%)	58/136 (42.6%)
**Immigrant**		
No	564/601 (93.8%)	173/564 (30.7%)
Yes	37/601 (6.2%)	10/37 (27.0%)
**Ever sex with women**		
No	255/601 (42.4%)	103/255 (40.4%)
Yes	346/601 (57.6%)	80/346 (23.1%)
**Sex in previous 3 months**		
No	23/601 (3.8%)	8/23 (34.8%)
Yes	578/601 (96.2%)	175/578 (30.3%)
**Sex with women in previous 3 months**		
No	484/600 (80.7%)	165/484 (34.1%)
Yes	116/600 (19.3%)	18/116 (15.5%)
**Anal sex with men in previous 3 months**		
No	52/600 (8.7%)	18/52 (34.6%)
Yes	548/600 (91.3%)	165/548 (30.1%)
**Ever tested for HIV**		
No	47/601 (7.8%)	12/47 (25.5%)
Yes	554/601 (92.2%)	171/554 (30.9%)
**Last HIV test**^a^		
Less than 1/2 year ago	345/596 (57.9%)	73/345 (21.2%)
6 months to a year ago	108/596 (18.1%)	41/108 (38.0%)
One year or longer ago	96/596 (16.1%)	52/96 (54.2%)
Never tested	47/596 (7.9%)	12/47 (25.5%)
**Number of prior HIV tests** ^a^		
Never	47/595 (7.9%)	12/47 (32.7%)
1–2 time	213/595 (35.8%)	84/213 (39.4%)
3 times and more	335/595 (56.3%)	82/335 (24.5%)
**Outcome of last HIV test (self-reported)**		
Positive	68/601 (11.3%)	67/68 (98.5%)
Negative	467/601 (77.7%)	93/467 (19.9%)
Don't know/no answer	14/601 (2.3%)	6/14 (42.9%)
Not applicable	52/601 (8.7%)	17/52 (32.7%)
**Study site**		
Kisumu, Kenya	169/601 (28.1%)	28/169 (16.6%)
Blantyre, Malawi	122/601 (20.3%)	27/122 (22.1%)
Cape Town, South Africa	139/601 (23.1%)	44/139 (31.7%)
Soweto, South Africa	171/601 (28.5%)	84/171 (49.1%)

^a^ Five participants who reported having had no prior HIV test, but had ARV drugs detected in a sample collected at the screening visit were classified as having had a prior HIV test; “Last HIV test” and “Number of prior HIV tests” were not known for these participants.

Abbreviations: MSM: men who have sex with men; TW: persons who identify as female or transgender; OR: odds ratio; CI: confidence intervals; AOR: adjusted odds ratio

**Fig 1:**
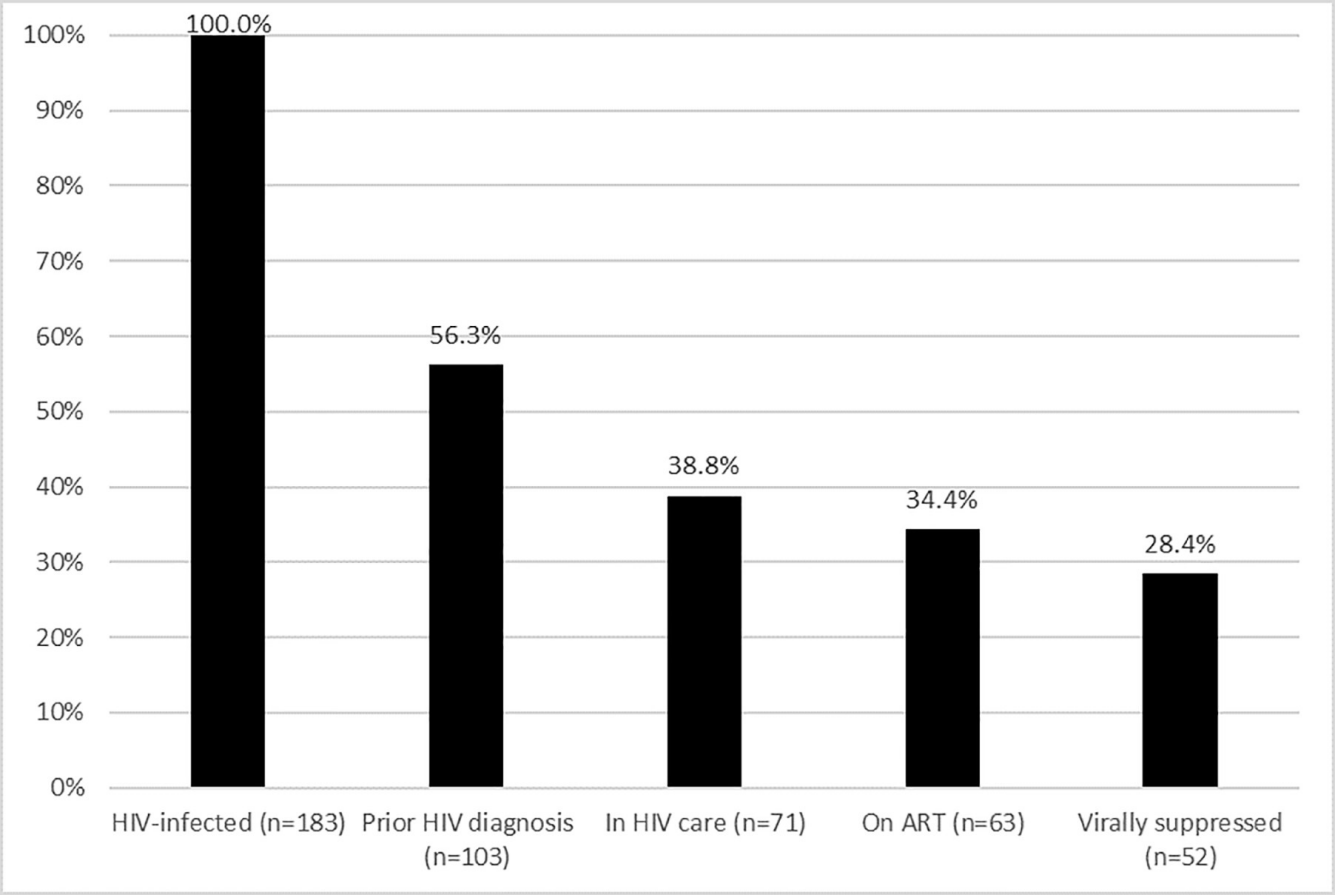
HIV care continuum for HIV-infected persons screened for participation in HPTN 075.

Forty-seven participants (7.8%; 47/601) were classified as never having had a prior HIV test (based on self-report and ARV drug testing). HIV testing at the screening visit revealed that 30.4% (183/601) of all participants were HIV infected. Among those who were HIV infected, 43.7% (80/183) were classified as newly diagnosed and 56.3% (103/183) were classified as previously diagnosed (based on self-report and the results of ARV drug testing) (Fig 1).

### Correlates of no prior HIV test

We compared the 47 (7.8%) MSM&TW who had no prior HIV test with the 554 (92.2%) MSM&TW who had a prior HIV test (Table 2). In univariate analyses, older age (21–25 years and 26–44 years) compared to younger age (18–20 years) was significantly associated with lower odds of never having had an HIV test. Living in Cape Town (South Africa) compared to living in Kisumu (Kenya) was significantly associated with higher odds of no prior HIV testing. (P<0.05 for all bivariate associations.) In multivariate analysis, older age (21–25 years and 26–44 years) compared to younger age (18–20 years) remained significantly associated with lower odds of no prior HIV testing (adjusted odds ratio [AOR]: 0.23, 95% confidence intervals [CI]: 0.11, 0.50; and AOR: 0.30, 95% CI: 0.14, 0.65, respectively).

**Table 2:** Characteristics of never having tested for HIV among MSM and TW screened for participation in HPTN 075 (N = 601)^a^.

		Univariate			Multivariate		
Variables	Never tested for HIV (%)	OR	95% CI	p-value	AOR	95% CI	p-value
**Age (years)**							
18–20	25/152 (16.4%)	REF					
21–25	10/237 (4.2%)	0.22	0.10, 0.48	0.0001	0.23	0.11, 0.51	0.0003
26–44	12/212 (5.7%)	0.30	0.15, 0.63	0.0013	0.30	0.14, 0.65	0.0020
**Gender identity**							
Male	36/465 (7.7%)	REF					
Transgender	11/136 (8.1%)	1.05	0.52, 2.12	0.8947			
**HIV status**							
Negative	35/418 (8.4%)	REF					
Positive	12/183 (6.6%)	0.77	0.39, 1.52	0.4467			
**Immigrant**							
No	46/564 (8.2%)	REF					
Yes	1/37 (2.7%)	0.31	0.04, 2.33	0.2570			
**Ever sex with women**							
No	24/255 (9.4%)	REF					
Yes	23/346 (6.6%)	0.69	0.38, 1.24	0.2142			
**Sex in previous 3 months**							
No	2/23 (8.7%)	REF					
Yes	45/578 (7.8%)	0.89	0.20, 3.90	0.8734			
**Sex with women in previous 3 months**							
No	35/484 (7.2%)	REF					
Yes	12/116 (10.3%)	1.48	0.74, 2.95	0.2649			
**Anal sex with men in previous 3 months**							
No	7/52 (13.5%)	REF					
Yes	40/548 (7.3%)	0.51	0.21, 1.19	0.1202			
**Study site**							
Kisumu, Kenya	7/169 (4.1%)	REF					
Blantyre, Malawi	12/122 (9.8%)	2.52	0.96, 6.61	0.0595	2.60	0.98, 6.91	0.0553
Cape Town, South Africa	15/139 (10.8%)	2.80	1.11, 7.07	0.0295	1.91	0.73, 4.97	0.1868
Soweto, South Africa	13/171 (7.6%)	1.90	0.74, 4.90	0.1815	1.64	0.63, 4.28	0.3119

^a^ Five men who reported having had no prior HIV test but had a positive HIV test result and tested positive for ARVs were recoded for this analysis as ever having tested for HIV.

Abbreviations: MSM: men who have sex with men; TW: persons who identify as female or transgender; OR: odds ratio; CI: confidence intervals; AOR: adjusted odds ratio; REF: reference value

Even though there was no significant difference between TW and cis-gender MSM, Table 3 shows that these groups differed in terms of the number of prior HIV tests and the self-reported result of their last HIV test. TW were less likely than MSM to have had 3 or more prior HIV tests. Despite this lower testing frequency, TW were more likely than MSM to report that their last HIV test was positive.

**Table 3:** HIV testing by gender identity among persons screened for participation in HPTN 075 (N = 601).

Variables	Transgender n/N (%)	Male n/N (%)	Chi^2^ *(p)*
**Ever tested for HIV**			0.02 (0.439)
No	11/136 (8.1)	36/465 (7.7)	
Yes	125/136 (91.9)	429/465 (92.3)	
**Last HIV test**^a^			6.59 (0.086)
Less than 1/2 year ago	74/133 (55.6)	271/463 (58.5)	
6 months to a year ago	18/133 (13.5)	90/463 (19.4)	
One year or longer ago	30/133 (22.6)	66/463 (14.3)	
Never tested	11/133 (8.3)	63/463 (7.8)	
**Number of prior HIV tests** ^a^			13.71 (0.001)
Never	11/133 (8.3)	36/462 (7.8)	
1–2 time	65/133 (48.9)*	148/462 (32.0)*	
3 times and more	57/133 (42.9)*	278/462 (60.2)*	
**Outcome of last HIV test (self-reported)**			14.01 (0.003)
Positive	26/136 (19.1)*	42/465 (9.0)*	
Negative	91/136 (66.9)*	376/465 (80.9)*	
Don't know/no answer	5/136 (3.7)	9/465 (1.9)	
Not applicable	14/136 (10.3)	38/465 (8.2)	

* Proportions that are significantly higher are lower than what would be expected based on marginal totals.

^a^ Five participants who reported having had no prior HIV test, but had ARV drugs detected in a sample collected at the screening visit were classified as having had a prior HIV test; “Last HIV test” and “Number of prior HIV tests” were not known for these participants.

Abbreviations: MSM: men who have sex with men; TW: persons who identify as female or transgender; OR: odds ratio; CI: confidence intervals; AOR: adjusted odds ratio.

### Correlates of HIV infection

We compared characteristics of the 183 (30.4%) HIV-infected MSM&TW and the 418 (69.6%) HIV-uninfected MSM&TW (Table 4). In univariate analyses, older age (26–44 years) was significantly associated with higher odds of being HIV infected compared to younger age (18–20 years). Identifying as female or transgender (compared to identifying as male), reporting having had an HIV test more than 6 months to one-year or ≥1 year prior to screening (compared to having had a test in the previous 6 months), and living in Cape Town or Soweto (South Africa; compared to living in Kisumu, Kenya) were significantly associated with higher odds of being HIV infected. Ever having had sex with women or having had sex with women in the past 3 months were significantly associated with lower odds of being HIV infected. (P<0.05 for all five variables.)

**Table 4:** Characteristics of HIV-infected vs. HIV-uninfected MSM and TW screened for participation in HPTN 075 (N = 601).

		Univariate			Multivariate^a^ ^b^		
Variables	HIV positive (%)	OR	95% CI	p-value	AOR	95% CI	p-value
**Age (years)**							
18–20	35/152 (23.0%)	REF					
21–25	66/237 (27.8%)	1.29	0.80, 2.07	0.2909	1.76	1.02, 3.02	0.0413
26–44	82/212 (38.7%)	2.11	1.32, 3.37	0.0018	3.18	1.81, 5.59	< .0001
**Gender identity**							
Male	125/465 (26.9%)	REF					
Transgender	58/136 (42.6%)	2.02	1.36, 3.01	0.0005	1.87	1.19, 2.95	0.0071
**Immigrant**							
No	173/564 (30.7%)	REF					
Yes	10/37 (27.0%)	0.84	0.40, 1.77	0.6409			
**Ever sex with women**							
No	103/255 (40.4%)	REF					
Yes	80/346 (23.1%)	0.44	0.31, 0.63	< .0001	0.64	0.40, 1.02	0.0620
**Sex in previous 3 months**							
No	8/23 (34.8%)	REF					
Yes	175/578 (30.3%)	0.81	0.34, 1.96	0.6457			
**Sex with women in previous 3 months**							
No	165/484 (34.1%)	REF					
Yes	18/116 (15.5%)	0.36	0.21, 0.61	0.0002	0.59	0.31, 1.10	0.0982
**Anal sex with men in previous 3 months**							
No	18/52 (34.6%)	REF					
Yes	165/548 (30.1%)	0.81	0.45, 1.48	0.5006			
**Last HIV test**^a^							
Less than 1/2 year ago	73/345 (21.2%)	REF					
6 months to a year ago	41/108 (38.0%)	2.28	1.43, 3.64	0.0005	2.08	1.25, 3.46	0.0048
One year or longer ago	52/96 (54.2%)	4.40	2.73, 7.10	< .0001	3.19	1.90, 5.38	< .0001
Never tested	12/47 (25.5%)	1.28	0.63, 2.58	0.4958	1.38	0.64, 2.97	0.4144
**Number of prior HIV tests**^a^							
Never	12/47 (25.5%)	REF					
1–2 times	84/213 (39.4%)	1.90	0.93, 3.87	0.0769			
3 times and more	82/335 (24.5%)	0.95	0.47, 1.91	0.8752			
**Study site**							
Kisumu, Kenya	28/169 (16.6%)	REF					
Blantyre, Malawi	27/122 (22.1%)	1.43	0.79, 2.58	0.2330	1.08	0.57, 2.03	0.8151
Cape Town, South Africa	44/139 (31.7%)	2.33	1.36, 4.00	0.0021	1.80	0.97, 3.33	0.0620
Soweto, South Africa	84/171 (49.1%)	4.86	2.94, 8.05	< .0001	3.40	1.90, 6.07	< .0001

^a^ Five men who reported no prior HIV test but had ARVs detected were excluded from this analysis because “Last HIV test” and “Number of prior HIV tests” were not known for these MSM.

^b^ To prevent overspecification, only covariates with p-value<0.05 in the univariate analysis were selected for multivariate analysis.

Abbreviations: MSM: men who have sex with men; TW: men who identify as female or transgender; OR: odds ratio; CI: confidence intervals; AOR: adjusted odds ratio.

In the multivariate analysis, significantly higher odds of being HIV infected were observed among participants 21–25 years and 26–44 years old (compared to 18–20 years; AOR: 1.76, 95% CI: 1.02, 3.02 and AOR: 3.18, 95% CI: 1.81, 5.59, respectively); participants who identified as female or transgender (compared to those identifying as male; AOR: 1.87, 95% CI: 1.19, 2.95); participants who reported that their last HIV test was 6–12 months earlier or ≥ 1 year earlier (compared to those reported having had an HIV test within the previous 6 months; AOR: 2.08, 95% CI: 1.25, 3.46 and AOR: 3.19, 95% CI: 1.90, 5.38, respectively); and participants in Soweto (South Africa) compared to those in Kisumu (Kenya; AOR: 3.40, 95% CI: 1.90, 6.07, respectively).

### Correlates of new HIV diagnoses

We compared the frequency and recency of prior HIV testing among MSM&TW classified as newly diagnosed (n = 80; 43.7%) vs. previously diagnosed (n = 103; 56.3%). In univariate analyses, being older and reporting more prior HIV tests (3 or more) were associated with lower odds of newly being diagnosed (Table 5). Significantly higher odds of being newly diagnosed were observed among participants who reported that their last HIV test was less recent; and among participants who lived in Blantyre (Malawi), Cape Town or Soweto (South Africa), compared to living in Kisumu (Kenya). (P<0.05 for all four variables.)

**Table 5:** Characteristics of MSM and TW with newly diagnosed vs. previously diagnosed HIV infections who were screened for participation in HPTN 075 (N = 183).

		Univariate			Multivariate^a^		
Variables	Newly diagnosed as HIV+ (%)	OR	95% CI	p-value	AOR	95% CI	p-value
**Age (years)**							
18–20	28/35 (80.0%)	REF			REF		
21–25	25/66 (37.9%)	0.15	0.06, 0.40	0.0001	0.19	0.06, 0.62	0.0056
26–44	27/82 (32.9%)	0.12	0.05, 0.32	< .0001	0.11	0.03, 0.39	0.0005
**Gender identity**							
Male	59/125 (47.2%)	REF					
Transgender	21/58 (36.2%)	0.63	0.33, 1.20	0.1644			
**Immigrant**							
No	77/173 (44.5%)	REF					
Yes	3/10 (30.0%)	0.53	0.13, 2.14	0.3754			
**Ever sex with women**							
No	50/103 (48.5%)	REF					
Yes	30/80 (37.5%)	0.64	0.35, 1.15	0.1361			
**Sex in previous 3 months**							
No	5/8 (62.5%)	REF					
Yes	75/175 (42.9%)	0.45	0.10, 1.94	0.2846			
**Sex with women in previous 3 months**							
No	76/165 (46.1%)	REF			REF		
Yes	4/18 (22.2%)	0.33	0.11, 1.06	0.0626	0.41	0.08, 2.10	0.2823
**Anal sex with men in previous 3 months**							
No	11/18 (61.1%)	REF					
Yes	69/165 (41.8%)	0.46	0.17, 1.24	0.1241			
**Last HIV test^a^**							
Less than 1/2 year ago	19/73 (26.0%)	REF			REF		
6 months to a year ago	23/41 (56.1%)	3.63	1.62, 8.15	0.0018	4.16	1.59,10.91	0.0037
One year or longer ago	26/52 (50.0%)	2.84	1.34, 6.04	0.0066	3.39	1.36, 8.46	0.0090
**Number of prior HIV tests ^a^**							
1–2 times	44/84 (52.4%)	REF			REF		
3 times and more	24/82 (29.3%)	0.38	0.20, 0.71	0.0028	0.66	0.31, 1.38	0.2671
**Study site**							
Kisumu, Kenya	5/28 (17.9%)	REF					
Blantyre, Malawi	14/27 (51.9%)	4.95	1.45,16.89	0.0106	6.76	1.44,31.79	0.0155
Cape Town, South Africa	26/44 (59.1%)	6.64	2.13,20.75	0.0011	2.60	0.55,12.23	0.2253
Soweto, South Africa	35/84 (41.7%)	3.29	1.14, 9.48	0.0278	2.04	0.49, 8.43	0.3249

^a^ Seventeen men who tested positive for HIV infection in the study but reported no prior HIV test were excluded from this analysis because “Last HIV test” and “Number of prior HIV tests” were not known for these participants.

Abbreviations: MSM: men who have sex with men; TW: persons who identify as female or transgender; OR: odds ratio; CI: confidence intervals; AOR: adjusted odds ratio; REF: reference value.

In multivariate analysis, significantly lower odds of being newly diagnosed were observed among MSM&TW who were older (21–25 years or 26–44 years compared to 18–20 years; AOR: 0.19, 95% CI: 0.06, 0.62 and AOR: 0.11, 95% CI: 0.03, 0.39, respectively). Significantly higher odds of being newly diagnosed were also observed among participants who reported that their last HIV test was 6–12 months or more than one year earlier, compared to those who reported that their last HIV test was <6 months earlier (AOR: 4.16, 95% CI: 1.59, 10.91; and AOR, 3.39; 95% CI: 1.36, 8.46, respectively). Finally, significantly higher odds of being newly diagnosed were observed among participants living in Blantyre (Malawi) compared to participants living in Kisumu (Kenya; AOR, 6.76; 95% CI: 1.44, 31.79).

## Care engagement and viral suppression

Of the 67 MSM&TW who reported that their last HIV test was positive and also tested positive at screening, 47.8% (32/67) reported that they were in care. The majority (81.3%, 26/32) of participants who reported to be in care also reported that ARV drugs had been prescribed. ARV drugs were detected in 88.5% (23/26) of the screening samples from these participants.

Of all participants who were previously diagnosed with HIV infection, 68.9% (71/103) were classified as being in care based on self-report and/or because ART drugs were detected (ARV drugs were detected in three participants who reported that their last HIV test was positive but reported that they were not in care, and in one person who reported that their last test was positive, who reported that they were in care, but said that ARV drugs had not been prescribed).

Of all newly- and previously-diagnosed HIV-infected participants screened for HPTN 075, 38.8% (71/183) were classified as being in care (Fig 1). ARV drugs were detected in samples from 88.7% (63/71) of those who were classified as in care; this corresponds to 61.2% (63/103) of the participants who were classified as previously diagnosed, and 34.4% (63/183) of all HIV-infected participants (Fig 1).

As reported earlier [43], HIV viral load was <400 copies/mL for 82.5% (52/63) of the participants who had ARV drugs detected; this corresponds to 47.7% (52/103) of the participants who were classified as previously diagnosed, and 28.4% (52/183) of all HIV-infected participants (Fig 1).

## Discussion

Although the majority of the MSM&TW screened for HPTN 075 reported that they had a prior HIV test, over a third reported that their last HIV test occurred more than 6 months prior to screening. Almost a third of all participants were HIV infected. The portion with HIV-infection was higher among older participants and among those who identified as female or transgender, even though testing frequency among TW was lower. Many of the HIV-infected participants were not aware of their positive HIV status; this was more likely the case among participants who reported that their last HIV test was less recent and among younger participants, who were also less likely to have had a prior HIV test. Almost a third of the participants who had previously been diagnosed with HIV infection reported that they were not engaged in care. The majority of the participants in care were on ART and the majority of those on ART were virally suppressed. The HIV care continuum gaps for HIV diagnosis and linkage to care explain why only 28.6% of all HIV-infected MSM&TW were virally suppressed (Fig 1).

Although the proportion of MSM&TW who reported no prior HIV test was low, there was a substantial number of previously undiagnosed HIV infections in this cohort. HIV prevalence among MSM&TW in this study was higher than reported HIV prevalence among men in the general African population in the same age group [5, 44]. The association of HIV infection with female or transgender identity (among those assigned the male sex at birth) that we observed has also been reported in other studies [2, 45, 46]. Awareness of one’s positive HIV status appears to be a major gap in the HIV care continuum in this population (Fig 1), especially for younger MSM&TW, who were the least likely to report having had a prior HIV test [47]. That participants who reported that their last HIV test was less recent had significantly higher odds of being newly diagnosed; this not surprising given the longer time interval, and indicates the importance of frequent and repeat testing in this population. The second biggest gap appears to be linkage to care among those with a prior HIV diagnosis (Fig 1). The remaining gaps were smaller in this study: many of those in care were on ART, and most of those on ART were virally suppressed. The differences we observed between study sites for HIV prevalence and prior HIV testing could reflect actual population differences (e.g., a higher general HIV prevalence in South Africa compared to Malawi); these differences may also reflect variations in the way persons were recruited at each study site.

This study cohort encompassed persons who were assigned male sex at birth and who identified as female or transgender, as well as cisgender MSM. This practice has been criticized for several reasons. First, the experiences of TW have not always been differentiated from those of MSM even though overlapping, they are distinctive [48, 49]. Furthermore, the comparison of TW with MSM perpetuates the notion that TW are men, and not women. As stated, we included TW in this study based on the experience that some TW in SSA identify with or see themselves as part of gay communities [23] and that HPTN 075 was designed to be inclusive and respect communities. Furthermore, the study allowed for the collection of exploratory information about the TW population in SSA, of which little is known. The findings from this study–elevated HIV prevalence and less frequent HIV testing among TW–strongly indicate that studying the health needs of TW in SSA in their own right is warranted.

A few limitations should be considered when interpreting the findings of this study. First, the number of potential correlates of HIV testing and care continuum outcomes that could be explored in this study was limited, because this analysis was based on data collected at screening. Second, some observed frequencies are small, resulting in large odds ratios and wide confidence intervals, affecting the statistical power and stability of the tested models. For instance, the odds of never having tested for HIV are higher in all sites compared to Kisumu, Kenya, but only the odds for Cape Town, South Africa are significant; it is likely that with a larger cohort, the other odds would have been significant as well. Third, the discrepancy between the self-reported HIV status and the results of ARV drug testing indicate that the accuracy of self-report is limited [42]. This discrepancy also indicates that the number of persons who were previously diagnosed with HIV infection but had not yet started ART, might actually be higher. Our rationale for analyzing self-reported data is that many HIV care and prevention programs rely on self-report when referring persons for HIV care or prevention services (most ARV assays are research-use only and are not generally available). The discrepancies we observed between self-reported data on HIV status and ARV drug detection highlight the need for more research to identify factors associated with the accuracy of self-reported data. Finally, findings from this study cannot be generalized to all MSM&TW in SSA because of the way in which persons were recruited. It is quite likely that the study recruited more easily accessible MSM&TW, who also might have had better access to HIV prevention and care services. This would imply that the actual status of the HIV testing and care continuum may be worse for African MSM&TW than the findings in this report suggest.

Our findings have several implications for programming and further research. These implications apply to both MSM and TW. However, implementation of these implications should acknowledge the differences between these populations. Furthermore, with the emerging transgender organizations in SSA [38], addressing TW in their own right seems warranted and feasible. In addition to promotion of safer sex practices and improved PrEP access and use, a critical step in preventing the further spread of HIV among MSM&TW in SSA is to ensure that persons are aware of their HIV status. Given the high prevalence of HIV infection among MSM&TW in this region and the various circumstances promoting risky sexual behavior, frequent repeat HIV testing is needed in this population. This is critical for all MSM&TW, but especially for younger MSM&TW. A second critical step in curbing the HIV epidemic in this population is to improve linkage to care for HIV-infected MSM&TW. Both steps require an understanding of barriers that operate on a personal and social level, such as anxiety about a positive diagnosis and HIV related stigma [50–53]. In addition, further information is needed about structural barriers to HIV testing and treatment, such as criminalization of homosexuality and limited access to appropriate health care [35, 54, 55]. Our findings suggest that once in care, gaps in the care continuum are less severe, but still need improvement. Several strategies have been proven effective for addressing these issues in other populations and settings [56–60]; adaptation of these strategies and evaluation of their effectiveness among SSA MSM&TW is urgently needed.

## Supporting information

S1 Dataset (CSV)

S1 Codebook (DOCX)
