# Supplementary material for: HIV testing and the HIV care continuum among sub-Saharan African men who have sex with men and transgender women screened for participation in HPTN 075
Source: PLoS One. 2019 May 31;14(5):e0217501. doi: 10.1371/journal.pone.0217501 (PMC6544251; doi:10.1371/journal.pone.0217501)
Supplement: S1 Codebook — (DOCX) [file pone.0217501.s002.docx]

| **Variable Names** | **Format** | **Description** | |  | |
| --- | --- | --- | --- | --- | --- |
| Uid |  | Participant uid | | | |
| sexwsex | 1="No"  3="Yes"  ; | Ever sex with women | | | |
| Sexlast | 1="More than 3 months ago"  2="Less than 3 months ago"  ; | Sex in previous 3 months | | | |
| Hivtimes_times | 0="Never "  1="1-2 times"  2="3 times and more"  ; | #number of times tested HIV  If HIVTEST=1 then HIVtimes_times=”never”  If HIVtimes=99 then HIVtimes_times=missing | | | |
| Hivlast | 1="Less than 1/2 year ago"  2="6 months to a year ago"  3="One year or longer ago"  ; | When was the last time that you got tested?  If HIVtest=1 then HIVLAST=missing |  | | |
| HTRfinal | 1="Negative"  2="Positive"  ; | HIV test result on screening | |  | |
| Siteid | 265 = "Kenya"  266 = "Malawi"  267 = "Cape Town"  268 = "Soweto"  ; | Study site | |  | |
| Derived_age |  | Participant age | |  | |
| d_age | 1="Age 18-20"  2="Age 21-25"  3="Age 26-44"  ; | Participant age group | |  | |
| Sexlastsex_r | 1= “No”  2= “Yes” | Had sex with women in last 3 months?  If sexlast=1 orsexlastsex=1 then sexlastsex_r=1;  If sexlastsex=2,3 then sexlastsex_r=2;  IF SEXLASTSEX=99 then sexlastsex_r=missing | |  | |
| Sexlasttype_anal | 1 = "Having had anal sex in previous 3 months"  0 = "Non-anal in previous 3 months"  ; | Anal sex with men in previous 3 months?  If sexlast=1 or sexlastsex=2 then sexlasttype_anal=0;  Else sexlasttype_anal=sexlasttype_3; | | |  |
| HIVreslt_r | 1="Positive"  2="Negative"  3="Don't know/no answer"  4="Not applicable" | Self-reported outcome of last HIV test  If HIVTEST=2, 99 the HIVreslt_r=4;  Else if hivreslt= 3, 99 then HIVreslt_r=3;  Else HIVrelst_r=hivreslt; | |  | |
| testhist | 1="Less than 1/2 year ago"  2="6 months to a year ago"  3="One year or longer ago"  4="Never tested"  ; | Testing history  If hivtest=2 then testhist=4;  Else testhist=hivlast; | |  | |
| Gender | 1 = "Transgender"  0 = "Male"  ; | Gender identification  If demgender_2=1 or demgender_3=1 or demgender_4_test in ("Transsexual" "Transvert" "Tranverstite" "partly male, partly female") then Gender=1;  else Gender=0; | |  | |
| Country | 0="No"  1="Yes"  ; | Immigrant  if DEMcountry=3 & siteid=268 then country=0;  else if DEMcountry=siteid-264 then country=0;  else if DEMcountry > 0 then country=1; | |  | |
| HIVtest2 | 0="No"  1="Yes"  ; | Ever tested HIV  hivtest2=2-hivtest; | |  | |
| Arv_use | 0= “ARV not tested”  1= “ARV tested”  ; | Tested ARV from LC | |  | |
| Aware_lc | 0= “newly diagnosed”  1= “not newly diagnosed” | Newly diagnosis status  if arv_use=1 or hivreslt2=1 then aware_lc=1;  else aware_lc=0; | |  | |
| Exclude_ana |  | Exclude_ana=1 need to be exclude for certain analysis. 5 men who reported noprior HIV test but had ARVs detected were excluded from this analysis because TESTING HISTORY and TESTING FREQUENCY were not known from these MSM. | |  | |
